# Accuracy of Optical Heart Rate Measurements for 10 Commercial Wearables in Different Climate Conditions and Activities: Instrument Validation Study

**DOI:** 10.2196/85186

**Published:** 2026-02-17

**Authors:** Jasper Gielen, Catharina Nina Van Oost, Glen Debard, Romy Sels, Nele A J De Witte, Toon Colman, Bert Bonroy, Jean-Marie Aerts

**Affiliations:** 1 Department of Biosystems, Research group M3-BIORES, KU Leuven, Kasteelpark Arenberg 30, Leuven, 3001, Belgium, +3216377112; 2 Mobilab & Care, Centre of Expertise Care and Well-being, Thomas More University of Applied Sciences, Geel, Belgium; 3 Psychology & Technology, Centre of Expertise Care and Well-being, Thomas More University of Applied Sciences, Antwerp, Belgium

**Keywords:** heart rate, wearable electronic devices, fitness trackers, mobile health, monitoring, exercise, stress, climate

## Abstract

**Background:**

Commercial wearable devices allow for continuous heart rate (HR) monitoring in daily life. Their accuracy under ecologically valid conditions, however, remains insufficiently independently tested, especially during irregular activity, cognitive stress, and variable climates.

**Objective:**

This study evaluated the HR accuracy of 10 commercially available wearables under controlled variations in physical activity, cognitive stress, and temperature. We hypothesized that physical activity irregularity, cognitive stress, and thermal climate conditions would affect measurement accuracy.

**Methods:**

Forty-five healthy adults (21‐68, mean 34, SD 12 y) completed a standardized protocol in climate-controlled chambers simulating neutral (23 °C), hot (36 °C), and cold (10 °C) conditions. Tasks included rest, cognitive stress (Montreal Imaging Stress Task), steady walking, and intermittent walking. Each of the 10 devices (Fitbit Charge 6, Fitbit Inspire 3, Garmin Vivosmart 5, Garmin Vivoactive 5, Apple Watch SE, Google Pixel Watch 2, Polar Ignite 3, Polar Pacer, Xiaomi Watch 2, and Oura Ring Gen 3) was compared against electrocardiogram-derived HR from a Zephyr BioHarness chest strap. Accuracy was assessed using mean absolute error (MAE), mean absolute percentage error (MAPE), repeated-measures concordance correlation coefficient (CCC), and Bland-Altman analysis.

**Results:**

Significant variability across the devices was observed. Fitbit Charge 6 (MAE 4.5 bpm, MAPE 5.5%, CCC 0.93) and Google Pixel Watch 2 (MAE 4.9 bpm, MAPE 6.7%, CCC 0.87) showed strong agreement with the gold standard. In contrast, Fitbit Inspire 3, Polar Ignite 3, Polar Pacer, and Oura Ring displayed larger errors (MAE 9‐14 bpm, MAPE 11%‐16%) and lower CCC values (0.45‐0.66). The climate conditions did not significantly affect the measurement accuracy of the test devices. The activity type, however, did have a significant effect: intermittent walking increased errors for multiple devices.

**Conclusions:**

Wearable HR measurement accuracy is device-specific and context-dependent. Moderate climates did not impair performance, but irregular movement reduced accuracy. Fitbit Charge 6 and Google Pixel Watch 2 demonstrated the highest reliability, supporting their use in health and sports monitoring. Careful device selection and context-aware interpretation remain critical for applied and clinical applications.

## Introduction

Commercial wearable devices have become increasingly embedded in everyday life, enabling continuous physiological monitoring across a range of real-world contexts. Optical heart rate (HR) monitoring via photoplethysmography (PPG) is now a standard feature in many wearable devices. These wearables offer the potential for unobtrusive health tracking, behavior change interventions, and remote monitoring of chronic conditions [1,2]. However, their practical utility hinges on the accuracy of the measurements they provide, especially in dynamic and ecologically valid conditions.

Despite the rapid expansion of the wearable technology market, the pace of independent scientific validation lags significantly behind device release cycles. Most commercial wearables are launched without peer-reviewed evidence supporting their measurement accuracy or reliability under diverse conditions [3-5]. This creates a critical gap between consumer expectations and clinical or research-grade data quality, particularly in domains such as health promotion, personalized exercise prescription, and digital health interventions [6].

HR is a core variable in wearable monitoring, often used as a proxy for different physiological processes such as physical exertion, emotional stress, or recovery status [7,8]. PPG-based HR measurements can, however, be affected by several external and physiological factors. These include motion artifacts from limb movement, which have been shown to lead to mean absolute percentage errors (MAPE) of over 20% during vigorous intensity exercise [9]. Sensor placement and poor contact can similarly introduce errors [10]. Variations in skin tone affect the absorption and scattering of the light used in PPG, which can contribute to errors. This has been shown in the past for darker-skinned users of different smartwatch brands that HR was underestimated by 10‐15 bpm at rest and by more than 20% during vigorous activity [11]. Finally, changes in blood flow due to vasoconstriction, vasodilation, or muscle tension also influence signal quality. For instance, significant changes in direct current and alternating current amplitudes of the PPG pulse have indicated that mild cold exposure has a substantial effect on finger blood circulation [12]. These authors suggested that mild cold exposure may have a delayed effect on the pulse transit time and, therefore, could be a potential source of error. Consequently, device performance may vary across activity types, from seated rest to vigorous or irregular movement, and may also be sensitive to environmental factors, such as temperature and humidity, which influence peripheral blood circulation [9].

To date, most validation studies of wearable HR monitoring have focused on standardized physical activities (eg, steady treadmill walking or cycling) under controlled laboratory conditions [13-15]. In these study designs, validation is performed across discrete, sustained activities, eg, walking at a certain intensity for 5 minutes. While such protocols provide valuable benchmarks, they often fail to capture the variability and unpredictability of everyday human movement. Moreover, few studies have systematically tested devices across a range of environmental conditions, despite growing interest in outdoor, occupational, or climate-sensitive applications of wearable monitoring [16]. Similarly, validation protocols rarely include cognitively demanding or stressful conditions, although wearables are increasingly applied for stress detection and mental health monitoring.

In this study, we evaluated the HR accuracy of 10 commercially available wearable devices across a standardized experimental protocol that varied in activity type, cognitive stress, and climate condition. The devices were selected based on their potential for use in research and commercial applications. The main selection criteria were cost, usability, and data availability.

Our goal was to assess how real-world influences, such as movement, cognitive stress, and ambient thermal environment, affect optical HR accuracy. We hypothesized that devices would show greater error during more vigorous and nonsteady-state activities due to increased motion artifacts. Additionally, acute cognitive stress was expected to reduce HR accuracy due to increased HR, cardiac contractility, systolic blood pressure, and peripheral vasoconstriction [17], all of which may affect PPG signal quality and accuracy [18]. Moreover, we hypothesized that exposure to extreme temperatures (hot or cold) would impair accuracy due to peripheral vasoconstriction or vasodilation affecting PPG signal quality.

## Methods

### Wearables

The following devices were included: Fitbit Charge 6, Fitbit Inspire 3, Garmin Vivosmart 5, Garmin Vivoactive 5, Apple Watch SE, Google Pixel Watch 2, Polar Ignite 3, Polar Pacer, Xiaomi Watch 2, and Oura (Gen 3). Thus, this selection contained 9 watches and 1 ring. The Zephyr BioHarness 3.0 chest strap was used as the gold standard reference measure. The Zephyr BioHarness 3.0 allows for collecting the raw electrocardiogram data and therefore to evaluate the data quality of the derived HR time series. It has demonstrated validity across many exercise modalities and contexts [19].

### Study Design and Participants

A total of 45 healthy adult volunteers (23 male, 22 female) participated in this study. The participants ranged in age from 21 to 68 years, with a mean age of 34 (SD 12) years. All participants were free from known cardiovascular, metabolic, or musculoskeletal conditions that could interfere with data collection or affect physiological responses. The participants completed an hour-long experimental protocol. During each session, participants wore 2 watches and 1 chest strap. To ensure that there was no optical or electromagnetic cross-interference, only 2 watches were worn at any time, 1 on the left wrist and 1 on the right wrist. The independent operation and data storage of all devices also mitigated any potential data interference. They were instructed to wear the smartwatch snugly, just above the wrist bone, ensuring the optical sensor maintained direct skin contact. In addition, 10 individuals wore the Oura ring on their nondominant hand. The ring (size 10) was worn on the finger that ensured stable sensor contact. Each of the 10 wearable devices was tested 10 times across different participants, yielding a total of 100 unique testing sessions (10 per device). To control for potential placement effects, the location of the devices was counterbalanced across the left and right hands. This sample size balanced device coverage against the logistical constraints of the multisession environmental protocol.

### Ethical Considerations

The study was approved by the Social and Societal Ethics Committee of KU Leuven (case G-2024-8195-R4(AMD)). Written informed consent was obtained from all study participants prior to participation after the nature and possible consequences of the studies were explained. To ensure privacy and confidentiality, all participant data were deidentified and stored on secure, password-protected institutional servers. No compensation was provided to participants.

### Experimental Protocol

All participants performed a standardized protocol in each of 3 environmental chambers: a neutral condition (23 °C, 50% relative humidity), a hot condition (36 °C, 70% relative humidity), and a cold condition (10 °C, 40% relative humidity). The 3 climate chambers were dimensionally identical (3.6 m by 2.4 m) and were located adjacent to one another, allowing for rapid participant transition. Half of the participants completed the protocol in the sequence neutral-hot-cold, while the other half followed the sequence neutral-cold-hot.

**Figure 1.**
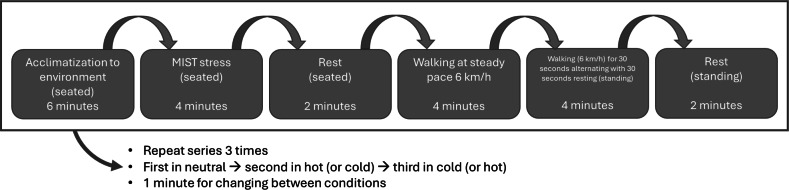
Timeline of the research protocol. MIST: Montreal Imaging Stress Task.

Within each environmental condition, participants completed six tasks lasting in total 22 minutes: (1) a 6-minute seated resting phase to acclimate to the chamber, (2) a 4-minute cognitive stress task (Montreal Imaging Stress Task [MIST] [20]) performed at a desk using a PC, (3) a 2-minute seated recovery phase, (4) a 4-minute steady walking task on a treadmill (Focus Fitness Jet 1) at 6 km/h, (5) a 4-minute intermittent walking task involving alternating 30-second walking and standing intervals, and (6) a final 2-minute standing rest phase. The sequence of these 6 tasks was fixed for all participants to maintain a standardized progression from rest to increasing cognitive and physical exertion, allowing for direct comparison of device performance across the controlled states. To mitigate systematic carry-over effects (eg, fatigue or learning) that might accumulate over the testing day, the order of the 3 main environmental conditions was systematically counterbalanced across participants. This experimental protocol was designed to simulate a range of ecologically valid use cases including cognitive and physical stressors, steady-state and intermittent movement, and environmental variation. The timeline of the research protocol is visualized in Figure 1.

### Data Collection and Processing

HR data from the Zephyr BioHarness and each wearable device were recorded continuously throughout all tasks. The data were exported using manufacturer-provided software platforms or application programming interfaces. This yielded the HR data at the highest possible sampling frequency for each device. The Polar Ignite 3, Polar Pacer, and Oura collected data at 1 Hz. For the other devices, the sampling frequency was variable with the intervals varying from 1 to 7 seconds. To ensure alignment, HR time series from each wearable were synchronized with the reference series using timestamp-based matching. Any reference data points that could not be matched due to sparse wearable sampling were excluded from analysis. No additional data smoothing or outlier removal was performed.

### Accuracy Metrics

Three key metrics were used to assess HR accuracy. First, mean absolute error (MAE) and MAPE were calculated between the wearable and reference HR signals for each condition and device. Second, we used the concordance correlation coefficient (CCC) [21]. Because the data consisted of repeated measurements over time for each participant, we extended the CCC using a repeated-measures framework [22]. The repeated-measures CCC accounts for the hierarchical structure of the data by estimating the variance components associated with between-subject and within-subject variability. This was done by fitting a linear mixed-effects model with a random intercept per participant, allowing variance components due to between-subject and within-subject variability to be separated. The repeated-measures CCC was then calculated using the model-based estimates of means, variances, and correlations, ensuring an unbiased agreement estimate under repeated measures. Third, we calculated the mean bias and limits of agreement for the Bland-Altman analysis [23] to assess the agreement between HR values recorded by the consumer wearable and the gold-standard chest strap. Similar to the calculation of the CCC, a mixed-effects adaptation of the original Bland-Altman analysis as described in Bland and Altman [24] was used to account for the repeated measurements.

The statistics outlined above were calculated both for the complete time series and for segments of the data corresponding to isolated activities and environmental conditions. This approach allowed for a statistical comparison to determine whether different conditions influenced the accuracy of the devices.

### Statistical Analysis

Before formal analysis, the normality of the MAE and MAPE values for the different subsets was tested using Shapiro-Wilk testing. The significance level (α) was set to .05 for these tests. Given that the null hypothesis of the normally distributed data was rejected in most cases, it was decided to apply robust nonparametric testing. To determine whether certain devices yielded significantly differing MAE and MAPE values, Kruskal-Wallis testing was performed. When the test yielded a significant result, multiple Wilcoxon rank sum tests were performed to identify which pairs of devices differ significantly. Similarly, it was studied whether the experimental conditions (ie, the type of activity or thermal environment) yielded significantly differing MAE and MAPE values for each device. For this purpose, Friedman testing was performed. When the test yielded a significant result, multiple Wilcoxon signed-rank tests were performed to identify which pairs of experimental conditions differed significantly. To account for the family-wise error rate of repeated testing, Bonferroni-corrected α-values were considered. Specifically, the Bonferroni correction was applied to all multiple pairwise Wilcoxon rank sum tests and Wilcoxon signed-rank tests to maintain a family-wise error rate of *α*=.05 across the multiple device and condition comparisons performed. To enhance the interpretability of statistically significant findings, we calculated and reported effect sizes for the pairwise nonparametric tests. For all pairwise Wilcoxon signed-rank and rank sum tests, the rank-biserial correlation (*r*) was used to quantify the magnitude of the difference. These metrics are indicated in the main text of the paper when presenting the results.

All analyses were performed in MATLAB using the *fitlme* function to fit mixed-effects models and custom scripts to compute the extracted parameters.

## Results

**Figure 2.**
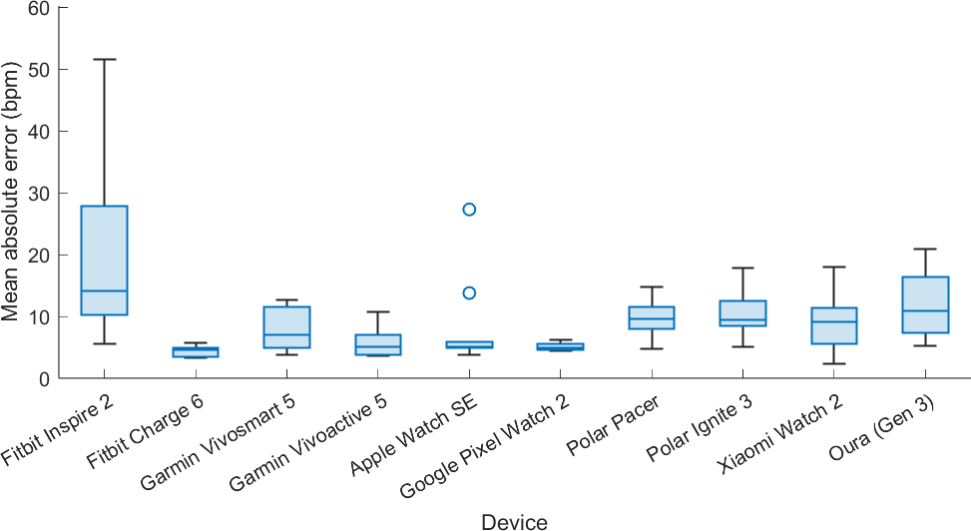
Visual representation of MAE values displayed in Table 1.

The statistics that describe the accuracy of the HR data for each wearable throughout the entire experimental protocol are summarized in Table 1. Furthermore, Figure 2 presents a visual comparison of the accuracy distributions (MAE values) across devices to allow for an immediate assessment of relative performance in addition to the tabulated values. The distribution of the MAE and MAPE values was used to test for significant differences between the various devices. The Kruskal-Wallis tests indicated that, for both the MAE and MAPE, at least 1 sample stochastically dominated one other sample (*P*<.05). In other words, there was a significant difference in the MAE and MAPE of at least 1 pair of wearables. Therefore, pairwise Wilcoxon rank sum tests were further performed to identify which pairs differ significantly. Given that the 10 wearables can form 45 unique pairs, the significance level was adjusted with the Bonferroni correction *(α*=.05/45=.0011). From these pairwise tests, it was observed that the MAE and MAPE values differed significantly between the Fitbit Charge 6 and the Fitbit Inspire 3, Polar Ignite 3, Polar Pacer, and Oura (Gen 3), with rank-biserial *r* values ranging between –0.76 and –0.84. In addition, significant differences for the MAE and MAPE were noted between the Fitbit Inspire 3 and the Garmin Vivoactive 5 and Google Pixel Watch 2 (rank-biserial *r* values between –0.75 and 0.84). Finally, the MAE values for the Google Pixel Watch 2 also differed significantly from the Polar Ignite 3 and Oura (Gen 3), with rank-biserial *r* values of –0.75 and –0.79, respectively.

**Table 1.** Measurement accuracy statistics for all tested wearables, over the full experimental protocol.

Wearable	MAE^a^ (bpm), median (IQR)	MAPE^b^ (%), median (IQR)	Repeated measures CCC^c^	Mixed-effects Bland-Altman		
				Mean bias (bpm)	Lower LoA^d^ (bpm)	Upper LoA (bpm)
Fitbit Inspire 3	14.3 (10.3-27.9)^e^	16.5 (11.1-40.6)^e^	0.45	–14.4	–51.3	22.5
Fitbit Charge 6	4.5 (3.5-5.0)^f^	5.5 (4.6-6.5)^f^	0.93	0.7	–11.2	12.7
Garmin Vivosmart 5	7.0 (4.9-11.5)	8.1 (6.5-15.6)	0.78	4.8	–15.8	25.4
Garmin Vivoactive 5	5.1 (3.8-7.15)	6.3 (5.5-7.5)	0.83	–1.0	–18.6	16.7
Apple Watch SE	5.0 (4.9-5.8)	7.3 (5.7-8.4)	0.70	0.9	–21.4	23.0
Google Pixel Watch 2	4.9 (4.5-5.6)^g^	6.7 (5.7-7.6)	0.87	–0.4	–15.0	14.2
Polar Ignite 3	9.5 (8.4-12.6)	11.2 (9.7-19.2)	0.63	–4.3	–31.4	22.9
Polar Pacer	9.7 (8.0-11.6)	13.1 (9.7-16.2)	0.66	–3.9	–29.7	21.8
Xiaomi Watch 2	9.1 (5.5-11.4)	11.9 (7.3-15.8)	0.69	–3.0	–33.3	27.2
Oura (Gen 3)	11.0 (7.3-16.4)	15.0 (8.3-20.8)	0.61	–7.1	–35.5	21.3

a MAE: mean absolute error.

b MAPE: mean absolute percentage error.

c CCC: concordance correlation coefficient.

d LoA: limits of agreement.

e Significantly different from Garmin Vivoactive 5 and Google Pixel Watch 2.

f Significantly different from Fitbit Inspire 3, Polar Ignite 3, Polar Pacer, and Oura (Gen 3).

g Significantly different from Polar Ignite 3 and Oura (Gen 3).

In addition to the comparison of the devices, we also evaluated the HR accuracy metrics for the different experimental conditions. Table 2 displays the accuracy of the HR data for each wearable in the 3 environmental conditions. Again, the distribution of the MAE and MAPE values was used to test for significant differences. In this case, it was checked for each wearable whether there were differences for the 3 climates. Given that 10 wearables were evaluated, the Friedman test was used 10 times. Therefore, the significance level was adjusted with the Bonferroni correction (*α*=.05/10=.005). The Friedman tests indicated that there were no significant differences in the device performance during the various climate conditions.

**Table 2.** Measurement accuracy statistics for all tested wearables in the 3 environmental conditions.

Environment wearable	MAE^a^, median (IQR)			MAPE^b^, median (IQR)			Repeated measures CCC^c^			Mixed-effects Bland-Altman								
	Neutral	Hot	Cold	Neutral	Hot	Cold	Neutral	Hot	Cold	Mean difference			Lower LoA^d^			Upper LoA		
										Neutral	Hot	Cold	Neutral	Hot	Cold	Neutral	Hot	Cold
Fitbit Inspire 3	9.6 (7.8-30.1)	20.8 (16.7-24.5)	10.6 (4.9-15.1)	11.4 (8.6-43.4)	24.4 (21.0-31.5)	10.1 (6.0-18.7)	0.52	0.58	0.36	–15.4	–10.5	–13.7	–49.2	–40.5	–51.4	18.5	19.5	24.0
Fitbit Charge 6	4.1 (3.4-4.6)	4.7 (4.0-5.4)	3.8 (3.0-4.1)	5.1 (4.3-5.8)	5.8 (4.9-6.6)	4.1 (3.4-6.0)	0.94	0.94	0.91	0.8	0.9	0.8	–10.2	–10.0	–12.5	11.9	11.8	14.0
Garmin Vivosmart 5	6.8 (5.4-10.8)	7.6 (5.6-15.9)	6.0 (5.1-7.1)	7.8 (6.1-13.6)	8.5 (7.0-20.7)	7.4 (5.4-8.9)	0.79	0.88	0.70	5.0	5.9	3.5	–14.4	–9.5	–20.6	24.5	21.2	27.6
Garmin Vivoactive 5	5.2 (3.7-5.7)	5.4 (4.4-7.5)	4.6 (3.3-6.2)	5.9 (4.7-6.8)	7.3 (6.2-9.9)	5.3 (4.8-7.4)	0.81	0.90	0.77	–0.6	–0.7	–1.5	–18.6	–14.6	–21.6	17.4	13.1	18.7
Apple Watch SE	5.0 (4.8-5.5)	5.6 (4.7-6.3)	5.0 (4.3-6.4)	7.1 (6.6-7.9)	7.6 (6.4-9.9)	6.8 (5.2-8.4)	0.83	0.71	0.75	2.5	–2.4	–0.8	–13.1	–23.8	–19.0	18.0	19.0	17.4
Google Pixel Watch 2	4.7 (4.3-5.5)	5.4 (4.8-6.2)	4.4 (4.1-5.6)	6.6 (5.9-7.4)	6.7 (6.3-8.8)	6.1 (4.4-7.5)	0.84	0.87	0.81	–0.6	0.2	–0.9	–14.7	–14.0	–16.0	13.5	14.4	14.2
Polar Ignite 3	8.7 (6.6-15.1)	13.9 (6.7-15.3)	6.1 (4.6-9.9)	9.5 (8.2-22.4)	18.2 (8.2-22.9)	9.2 (5.7-15.2)	0.59	0.75	0.55	–2.7	–2.4	–7.7	–29.6	–23.2	–37.5	24.2	18.5	22.2
Polar Pacer	8.4 (5.4-10.6)	13.8 (5.9-17.1)	7.5 (5.4-7.8)	9.9 (7.9-17.6)	17.3 (7.4-26.8)	9.6 (6.3-11.4)	0.67	0.78	0.52	–3.6	–2.3	–7.2	–26.8	–21.2	–37.8	19.5	16.5	23.4
Xiaomi Watch 2	6.1 (3.5-10.4)	9.5 (7.0-22.5)	5.3 (4.1-9.4)	7.8 (4.6-12.7)	12.7 (8.0-20.7)	7.3 (4.2-10.1)	0.81	0.86	0.56	–2.1	–2.2	–4.9	–20.9	–20.5	–44.8	16.7	16.2	34.9
Oura (Gen 3)	9.4 (4.6-11.0)	16.2 (11.4-20.7)	9.1 (5.4-11.3)	9.9 (5.6-13.9)	18.3 (13.8-26.7)	10.5 (5.1-18.2)	0.78	0.80	0.32	–5.5	–7.2	–9.3	–25.3	–27.6	–40.5	14.2	13.5	21.9

a MAE: mean absolute error.

b MAPE: mean absolute percentage error.

c CCC: concordance correlation coefficient.

d LoA: limits of agreement.

Similar to testing for climate-related differences, the HR accuracy metrics were also checked for differences between the 3 types of activity: performing the MIST test, walking at a steady pace, and walking intermittently. Table 3 displays the statistics of the HR data for each wearable during the 3 activities. The Friedman test was again repeated for each wearable, and thus the Bonferroni was corrected to an *α* of .005. As a result, the tests indicated significant differences for the Apple Watch SE, Google Pixel Watch 2, Polar Ignite 3, and Xiaomi Watch 2. Therefore, multiple Wilcoxon signed rank tests were further performed to identify which pairs differed significantly, with rank-biserial *r* values ranging between 0.82 and 0.97. The Bonferroni-corrected significance level was set (*α*=.05/3=.0167) for the devices that are each tested for the 3 activities. The bilateral differences are indicated in Table 3.

**Table 3.** Measurement accuracy statistics for all tested wearables for the 3 activities performed.

Activity wearable	MAE^a^, median (IQR)			MAPE^b^, median (IQR)			Repeated measures CCC^c^			Mixed-effects Bland-Altman								
	MIST^d^	Walking	Walking intermittent	MIST	Walking	Walking intermittent	MIST	Walking	Walking intermittent	Mean difference			Lower LoA^e^			Upper LoA		
										MIST	Walking	Walking intermittent	MIST	Walking	Walking intermittent	MIST	Walking	Walking intermittent
Fitbit Inspire 3	7.3 (4.2-18.3)	10.5 (6.8-21.4)	12.1 (7.1-17.4)	10.1 (6.3-27.2)	11.0 (6.6-23.0)	14.5 (6.4-18.5)	0.12	0.16	0.29	–11.1	–5.7	–13.8	–41.3	–33.3	–37.1	19.2	21.9	9.4
Fitbit Charge 6	3.6 (3.0-4.5)	3.8 (2.9-4.2)	3.4 (2.8-4.3)	5.2 (4.0-6.0)	3.7 (3.2-4.5)	3.6 (3.0-5.5)	0.67	0.78	0.80	0.8	3.3	0.5	–8.8	–8.3	–9.3	10.4	15.0	10.4
Garmin Vivosmart 5	5.3 (4.1-6.2)	7.9 (5.7-15.8)	6.5 (5.4-19.2)	7.4 (6.0-8.4)	6.5 (5.8-16.8)	6.8 (5.2-25.4)	0.42	0.41	0.34	6.9	7.1	0.7	–5.7	–17.6	–17.6	19.5	31.8	19.1
Garmin Vivoactive 5	4.3 (2.8-5.3)	5.9 (3.9-9.8)	5.6 (3.5-6.7)	5.4 (4.6-7.2)	6.4 (4.2-9.1)	5.9 (4.5-7.6)	0.49	0.55	0.52	0.6	0.6	–3.4	–11.7	–22.1	–20.1	12.8	23.2	13.4
Apple Watch SE	4.3^f^ (3.6-6.4)	4.5^f^ (4.0-6.9)	6.3 (5.4-7.6)	6.8 (4.8-9.0)	5.0^f^ (3.8-7.6)	8.6 (6.3-9.3)	0.29	0.49	0.28	1.6	–0.8	–0.9	–10.0	–24.4	–28.5	13.3	22.7	26.6
Google Pixel Watch 2	4.0 (3.7-4.5)	6.1^g^ (5.2-10.4)	5.5^g^ (4.5-5.7)	6.2 (4.3-7.7)	6.2 (5.5-9.0)	6.8 (4.6-7.4)	0.63	0.45	0.72	0.1	–0.1	–1.0	–10.3	–22.2	–13.8	10.4	22.1	11.8
Polar Ignite 3	5.9 (3.3-6.8)	11.8 (7.2-16.8)	12.0^g^ (10.3-16.8)	9.1 (4.6-9.6)	13.0 (7.2-14.3)	13.8^g^ (10.7-23.4)	0.30	0.21	0.08	–1.0	2.9	–12.6	–15.6	–25.4	–37.6	13.7	31.1	12.3
Polar Pacer	7.0 (3.4-10.7)	8.2 (5.4-9.4)	10.5 (6.6-17.1)	10.4 (4.8-12.8)	7.9 (6.0-11.0)	11.4 (7.3-20.8)	0.21	0.23	0.18	–2.2	2.7	–8.5	–20.7	–21.0	–31.3	16.2	26.4	14.4
Xiaomi Watch 2	4.7 (2.9-7.3)	11.1^g^ (6.4-14.1)	10.0^g^ (8.0-19.2)	7.5 (4.3-10.2)	12.3^g^ (6.0-15.7)	11.2^g^ (8.2-21.1)	0.40	0.36	0.20	–1.1	1.2	–6.8	–15.7	–38.8	–42.2	13.4	41.2	28.5
Oura (Gen 3)	8.0 (4.2-20.4)	9.3 (4.7-13.9)	7.0 (5.1-13.5)	11.5 (5.1-29.0)	10.1 (4.7-14.0)	8.7 (4.3-16.4)	0.13	0.36	0.38	–7.8	1.3	–6.0	–33.7	–22.8	–25.9	18.2	25.4	13.9

a MAE: mean absolute error.

b MAPE: mean absolute percentage error.

c CCC: concordance correlation coefficient.

d MIST: Montreal Imaging Stress Task.

e LoA: limits of agreement.

f Significantly different from walking intermittently.

g Significantly different from performing the Montreal Imaging Stress Task test.

## Discussion

### Principal Results

This study evaluated the HR accuracy of 10 commercially available wearable devices (9 watches and 1 ring) under varying environmental and activity conditions. Accuracy was assessed using multiple statistical approaches, including absolute and relative error metrics (MAE and MAPE), concordance with a reference chest strap (repeated-measures CCC), and agreement intervals (Bland-Altman analysis). To interpret these outcomes, established standards were applied. For example, the American National Standards Institute specifies a boundary of ±10% MAPE or ±5 bpm, whichever is greater, as acceptable error for cardiac monitors [25]. Correlation coefficients were interpreted as weak (≤0.5), moderate (0.5‐0.7), or strong (≥0.7) [26,27], while a stricter criterion of CCC ≥0.80 has also been proposed in previous work [13,28]. Within this framework, our findings indicate differences in device performance: the Fitbit Inspire 3 and Oura Ring exhibited significantly higher errors and wider limits of agreement, whereas the Fitbit Charge 6 and Google Pixel Watch 2 met both the criterion of CCC ≥0.80 and the American National Standards Institute error boundaries. Interestingly, these results demonstrate that accuracy is not only brand-dependent but also device-specific, as even wearables from the same manufacturer, despite relying on similar PPG technology, can yield different outcomes.

Participants underwent repeated testing across 3 thermal environments, neutral (23 °C), hot (36 °C), and cold (10 °C), but we found no statistically significant effect of temperature condition on device accuracy after the Bonferroni correction. However, the type of physical activity did significantly affect HR measurement accuracy in several devices. Specifically, walking intermittently elicited larger errors in a subset of wearables. These results support our initial hypothesis that motion irregularity is a strong determinant of PPG-based HR accuracy.

### Comparison With the State of the Art

The significant variability in accuracy across commercial wearables is consistent with earlier validation studies. Düking et al [29], for example, found marked differences in HR accuracy among 4 commercial wrist-worn devices (Apple Watch 4, Polar Vantage V, Garmin Fenix 5, and Fitbit Versa) during a structured treadmill protocol. They observed that vigorous or intermittent activities, particularly those involving sudden directional changes, were prone to introducing motion artifacts that degraded PPG signal quality. Our findings extend this line of work by demonstrating that even seemingly modest variations in movement patterns (eg, intermittent walking) can impact accuracy and that these effects differ between devices.

Interestingly, we did not find significant accuracy differences between climate conditions, which contradicts some theoretical expectations and anecdotal observations. Cold-induced vasoconstriction and heat-induced vasodilation are known to affect peripheral blood flow and optical signal amplitude [3,8,30]. However, it is possible that our selected temperature ranges (10-36 °C) were not extreme enough to meaningfully affect PPG signal quality, particularly in a short-term indoor protocol. Alternatively, modern signal-processing algorithms embedded in the wearables may have compensated for these physiological variations.

Prior studies have also highlighted the limitations of wrist-worn devices for certain populations and use cases. For example, variability in skin tone, arm hair, or wrist circumference can influence optical HR signal integrity [13,31], and these factors were not explicitly accounted for in our study. Nevertheless, the consistency of our findings across participants and the robustness of the repeated-measures statistical approach support the generalizability of our device comparisons.

Notably, the Fitbit Charge 6 consistently showed high accuracy across all conditions, with low MAE, low MAPE, and high repeated-measures CCC (>0.90). The Google Pixel Watch 2 and Garmin Vivosmart 5 also performed well, particularly during steady-state walking. In contrast, the Polar Ignite 3, Polar Pacer, and Oura Ring exhibited wider limits of agreement and lower CCC values, particularly during irregular activity. These differences may reflect varying PPG sensor designs, sampling rates, and proprietary signal-cleaning algorithms [32,33].

### Physiological and Technical Considerations

PPG is inherently sensitive to movement artifacts, particularly when motion occurs along the axis of the light source or in tissues with variable compressibility (eg, wrists with varying musculature) [34]. Devices such as the Oura Ring, which is worn on the finger, may face additional challenges due to the vasomotor sensitivity of the digits and the smaller optical contact area. While rings offer the advantage of less wrist movement, they may be more susceptible to temperature-driven changes in blood flow [35], which could explain the relatively poorer performance of the Oura device in this study.

Furthermore, activity context plays a significant role. The MIST task, a standardized cognitive stressor with minimal physical exertion, was associated with lower HR variability and motion. This is consistent with evidence that cognitive demands and psychological stress typically reduce HR variability [36]. As a result, wearables generally performed well under these conditions. In contrast, intermittent walking introduced frequent postural and muscular transitions, posing a challenge for devices dependent on motion-compensated PPG. These findings align with previous observations that accuracy degrades in nonsteady-state conditions and that current algorithms may overfit to predictable, repetitive activity patterns [37,38].

### Study Limitations

Several limitations should be acknowledged. First, the duration of each activity phase was relatively short (4‐6 min). Longer monitoring periods might have revealed delayed effects of thermal stress or device drift. Second, while we designed the environmental conditions to simulate realistic hot and cold exposures, extreme climates or outdoor settings were not replicated. Third, we did not directly measure skin temperature or blood flow perfusion, which could have elucidated underlying physiological causes of error.

Moreover, our participant sample, although balanced across devices and arms, did not include diverse skin tones or body compositions, which may limit generalizability. We note from the literature that variations in skin tone are known to bias PPG accuracy [11], suggesting that device performance may differ in diverse populations. The skin tone of the participants in our study was not included in the data collection. In hindsight, we note a relatively homogenous population of lighter skin phototypes (Fitzpatrick type I-III). Additionally, while the sample had a broad age range (21‐68 y) and a balanced sex distribution, the sample was predominantly younger adults, and only 5 older participants (ages 47‐68) were involved. Hence, a formal analysis to quantify the isolated impact of skin tone, age, and sex on device accuracy was not performed, which may be a focus for future research. Finally, as with any study of commercial wearables, device firmware and algorithms may change over time, making it difficult to extrapolate our results to future updates of the same models.

### Implications for Research and Practice

Our findings offer practical insights for researchers and health professionals selecting wearable devices for HR monitoring in mixed conditions. Devices such as the Fitbit Charge 6 and Google Pixel Watch 2 may offer sufficient accuracy for light-to-moderate physical activities in typical environmental conditions. However, researchers should exercise caution when using wearables for irregular activities or for applications that require high precision (eg, clinical monitoring or dose-response studies).

The study protocol can easily be replicated with other wearable devices, enabling comparisons between new devices and the 10 devices that have already been evaluated. For future validation studies, the inclusion of diverse populations, extended durations, and wearable signal-quality indices (eg, raw PPG, accelerometer-derived movement indices) would allow for a more granular understanding of error sources. Open-science practices, including the publication of raw validation data and code for statistical methods, will further support reproducibility and transparency in wearable health technology research.

### Conclusions

In this comparative study of 10 commercial wearable devices, significant differences in optical HR accuracy were observed between brands and activity types. Climate conditions did not produce statistically significant effects on accuracy, suggesting robustness within the moderate temperature ranges tested. However, irregular movement patterns degraded performance in multiple wearables, highlighting the need for careful selection and context-aware interpretation of HR data in applied health and sports settings. Devices such as the Fitbit Charge 6 and Google Pixel Watch 2 demonstrated strong agreement with reference chest strap measurements, even across varied protocols. The device-specific findings should be interpreted with caution, as they pertain to the investigated models. Accuracy may vary considerably across other devices within the same brand, despite the use of similar PPG technology.

## Abbreviations

CCC: concordance correlation coefficient
HR: heart rate
MAE: mean absolute error
MAPE: mean absolute percentage error
MIST: Montreal Imaging Stress Task
PPG: photoplethysmography

## References

[1] Dinh-Le C, Chuang R, Chokshi S, Mann D (2019). Wearable health technology and electronic health record integration: scoping review and future directions. JMIR Mhealth Uhealth.

[2] Abd-Alrazaq A, AlSaad R, Shuweihdi F, Ahmed A, Aziz S, Sheikh J (2023). Systematic review and meta-analysis of performance of wearable artificial intelligence in detecting and predicting depression. NPJ Digit Med.

[3] Bent B, Goldstein BA, Kibbe WA, Dunn JP (2020). Investigating sources of inaccuracy in wearable optical heart rate sensors. NPJ Digit Med.

[4] Evenson KR, Goto MM, Furberg RD (2015). Systematic review of the validity and reliability of consumer-wearable activity trackers. Int J Behav Nutr Phys Act.

[5] Peake JM, Kerr G, Sullivan JP (2018). A critical review of consumer wearables, mobile applications, and equipment for providing biofeedback, monitoring stress, and sleep in physically active populations. Front Physiol.

[6] Dobson R, Stowell M, Warren J (2023). Use of consumer wearables in health research: issues and considerations. J Med Internet Res.

[7] Shaffer F, Ginsberg JP (2017). An overview of heart rate variability metrics and norms. Front Public Health.

[8] Buchheit M (2014). Monitoring training status with HR measures: do all roads lead to Rome?. Front Physiol.

[9] Fine J, Branan KL, Rodriguez AJ (2021). Sources of inaccuracy in photoplethysmography for continuous cardiovascular monitoring. Biosensors (Basel).

[10] Vermunicht P, Buyck C, Naessens S (2025). Optimization and pre-use suitability selection for wrist photoplethysmography-based heart rate monitoring in patients with cardiac disease. Eur Heart J Digit Health.

[11] Asif S, AlSaafeen A, Nadar S (2025). Photoplethysmography in diverse skin tones: evaluating bias in smartwatch health monitoring. Cureus.

[12] Zhang XY, Zhang YT (2006). The effect of local mild cold exposure on pulse transit time. Physiol Meas.

[13] Gillinov S, Etiwy M, Wang R (2017). Variable accuracy of wearable heart rate monitors during aerobic exercise. Med Sci Sports Exerc.

[14] Stahl SE, An HS, Dinkel DM, Noble JM, Lee JM (2016). How accurate are the wrist-based heart rate monitors during walking and running activities? Are they accurate enough?. BMJ Open Sport Exerc Med.

[15] Kroll RR, Boyd JG, Maslove DM (2016). Accuracy of a wrist-worn wearable device for monitoring heart rates in hospital inpatients: a prospective observational study. J Med Internet Res.

[16] Falter M, Budts W, Goetschalckx K, Cornelissen V, Buys R (2019). Accuracy of apple watch measurements for heart rate and energy expenditure in patients with cardiovascular disease: cross-sectional study. JMIR Mhealth Uhealth.

[17] Carter JR, Kupiers NT, Ray CA (2005). Neurovascular responses to mental stress. J Physiol.

[18] Tamura T, Maeda Y, Sekine M, Yoshida M (2014). Wearable photoplethysmographic sensors—past and present. Electronics (Basel).

[19] Nazari G, Bobos P, MacDermid JC, Sinden KE, Richardson J, Tang A (2018). Psychometric properties of the Zephyr bioharness device: a systematic review. BMC Sports Sci Med Rehabil.

[20] Dedovic K, Renwick R, Mahani NK, Engert V, Lupien SJ, Pruessner JC (2005). The Montreal Imaging Stress Task: using functional imaging to investigate the effects of perceiving and processing psychosocial stress in the human brain. J Psychiatry Neurosci.

[21] Lin LI (1989). A concordance correlation coefficient to evaluate reproducibility. Biometrics.

[22] Carrasco JL, King TS, Chinchilli VM (2009). The concordance correlation coefficient for repeated measures estimated by variance components. J Biopharm Stat.

[23] Bland JM, Altman DG (1986). Statistical methods for assessing agreement between two methods of clinical measurement. Lancet.

[24] Bland JM, Altman DG (2007). Agreement between methods of measurement with multiple observations per individual. J Biopharm Stat.

[25] Association for the Advancement of Medical Instrumentation, American National Standards Institute (1995). AAMI Standards and Recommended Practices Volume 22: Biomedical Equipment Part 2: Monitoring and Diagnostic Equipment.

[26] Nelson BW, Allen NB (2019). Accuracy of consumer wearable heart rate measurement during an ecologically valid 24-hour period: intraindividual validation study. JMIR Mhealth Uhealth.

[27] Wallen MP, Gomersall SR, Keating SE, Wisløff U, Coombes JS (2016). Accuracy of heart rate watches: implications for weight management. PLoS ONE.

[28] Pasadyn SR, Soudan M, Gillinov M (2019). Accuracy of commercially available heart rate monitors in athletes: a prospective study. Cardiovasc Diagn Ther.

[29] Düking P, Giessing L, Frenkel MO, Koehler K, Holmberg HC, Sperlich B (2020). Wrist-worn wearables for monitoring heart rate and energy expenditure while sitting or performing light-to-vigorous physical activity: validation study. JMIR Mhealth Uhealth.

[30] Zhang Z (2015). Photoplethysmography-based heart rate monitoring in physical activities via joint sparse spectrum reconstruction. IEEE Trans Biomed Eng.

[31] Hung SH, Serwa K, Rosenthal G, Eng JJ (2021). Validity of heart rate measurements in wrist-based monitors across skin tones during exercise. PLOS ONE.

[32] Wang R, Blackburn G, Desai M (2017). Accuracy of wrist-worn heart rate monitors. JAMA Cardiol.

[33] Parak J, Korhonen I Evaluation of wearable consumer heart rate monitors based on photopletysmography.

[34] Spierer DK, Rosen Z, Litman LL, Fujii K (2015). Validation of photoplethysmography as a method to detect heart rate during rest and exercise. J Med Eng Technol.

[35] Pilt K, Meigas K, Temitski K, Viigimaa M The effect of local cold and warm exposure on index finger photoplethysmographic signal waveform.

[36] Hansen AL, Johnsen BH, Thayer JF (2003). Vagal influence on working memory and attention. Int J Psychophysiol.

[37] Mühlen JM, Stang J, Lykke Skovgaard E (2021). Recommendations for determining the validity of consumer wearable heart rate devices: expert statement and checklist of the INTERLIVE Network. Br J Sports Med.

[38] Van Oost CN, Masci F, Malisse A (2025). Accuracy of heart rate measurement under transient states: a validation study of wearables for real-life monitoring. Sensors (Basel).

[39] Suchikova Y, Tsybuliak N, Teixeira da Silva JA, Nazarovets S (2025). GAIDeT (Generative AI Delegation Taxonomy): a taxonomy for humans to delegate tasks to generative artificial intelligence in scientific research and publishing. Account Res.

